# Mobile Phone Cognitive Bias Modification Research Platform for Substance Use Disorders: Protocol for a Feasibility Study

**DOI:** 10.2196/resprot.9740

**Published:** 2018-06-12

**Authors:** Melvyn Zhang, JiangBo Ying, Guo Song, Daniel SS Fung, Helen Smith

**Affiliations:** ^1^ National Addictions Management Service Institute of Mental Health Singapore Singapore; ^2^ Department of Developmental Psychiatry Institute of Mental Health Singapore Singapore; ^3^ Family Medicine and Primary Care Lee Kong Chian School of Medicine Nanyang Technological University Singapore Singapore Singapore

**Keywords:** attention bias modification, eHealth, mHealth, development

## Abstract

**Background:**

Cognitive biases refer to automatic attentional and interpretational tendencies, which could be retained by cognitive bias modification interventions. Cristea et al and Jones et al have published reviews (in 2016 and 2017 respectively) on the effectiveness of such interventions. The advancement of technologies such as electronic health (eHealth) and mobile health (mHealth) has led to them being harnessed for the delivery of cognitive bias modification. To date, at least eight studies have demonstrated the feasibility of mobile technologies for the delivery of cognitive bias modification. Most of the studies are limited to a description of the conventional cognitive bias modification methodology that has been adopted. None of the studies shared the developmental process for the methodology involved, such that future studies could adopt it in the cost-effective replication of such interventions.

**Objective:**

It is important to have a common platform that could facilitate the design and customization of cognitive bias modification interventions for a variety of psychiatric and addictive disorders. It is the aim of the current research protocol to describe the design of a research platform that allows for customization of cognitive bias modification interventions for addictive disorders.

**Methods:**

A multidisciplinary team of 2 addiction psychiatrists, a psychologist with expertise in cognitive bias modification, and a computer engineer, were involved in the development of the intervention. The proposed platform would comprise of a mobile phone version of the cognitive bias task which is controlled by a server that could customize the algorithm for the tasks and collate the reaction-time data in realtime. The server would also allow the researcher to program the specific set of images that will be present in the task. The mobile phone app would synchronize with the backend server in real-time. An open-sourced cross-platform gaming software from React Native was used in the current development.

**Results:**

Multimedia Appendix 1 contains a video demonstrating the operation of the app, as well as a sample dataset of the reaction times (used for the computation of attentional biases) captured by the app.

**Conclusions:**

The current design can be utilized for cognitive bias modification across a spectrum of disorders and is not limited to one disorder. It will be of value for future research to utilize the above platform and compare the efficacy of mHealth approaches, such as the one described in this study, with conventional Web-based approaches in the delivery of attentional bias modification interventions.

**Registered Report Identifier:**

RR1-10.2196/9740

## Introduction

Cognitive biases include automatic attentional and interpretational tendencies [1]. Attentional biases, which refer to the preferential tendency for attention to be allocated towards or away from stimuli that are emotionally salient for the individual, have been found to be present in a variety of disorders, ranging from depression and anxiety disorders to addictive disorders [2]. These automatic processes would result in individuals with an anxiety disorder to attend to threat related cues, individuals with depression to attend to negative information, and individuals with addictive disorders to attend to substance-related cues. As such, cognitive biases are highly prevalent and have been implicated in the psychopathologies of various disorders [3-5].

Various theoretical approaches have helped to account for the role of these automatic processes in the different psychopathologies, but, perhaps the best developed theoretical model to date, is the dual-process model for addictive disorders [6]. The dual-process theoretical model proposes that with the recurrent use of a substance, this facilitates increased automatic processing of substance-related cues, with a resultant inhibition of the normal cognitive control processes [6]. Along with the increased recognition of cognitive biases, there has been further research examining how these biases could be modified. Several different cognitive bias modification approaches are routinely used, namely that of the attention bias modification, cognitive bias modification for interpretations, spatial cueing tasks, and attentional visual search [7]. To date, there have been a number of studies reported that have investigated the effectiveness of bias modification. In 2016, Cristea et al conducted a meta-analysis of participants with either a tobacco or alcohol addiction and considered the effect size of cognitive bias modification on cognitive biases and on addiction-related outcomes, such as cravings [8]. This study reported that bias modification had a significant effect on cognitive bias, with an effect size of 0.60 (calculated using Hedge g). Despite its effect on cognitive biases, there were no effects reported on other addiction outcomes. In a commentary published online, responding to the study by Cristea et al [8], a key limitation of the meta-analysis was identified, namely that Cristea et al considered clinical and nonclinical trials jointly in their synthesis of the results. When considering only clinical trials, there was a significant effect of bias modification. This finding from the meta-analysis demonstrates the importance of targeting automatic processes and the potential efficacy of bias modification in substance using individuals. A review of meta-analyses published by Jones et al in 2017 [1], conducted for a diverse group of participants, reported that the current evidence supports the effectiveness of bias modification, mainly for anxiety disorders.

Conventional cognitive bias interventions have typically been delivered using a computer in the confines of a laboratory, but there is potential to change this. In the past decade, there have been an increasing number of remote online therapies available and this has been attributed to the advances in eHealth, or electronic health. eHealth technologies facilitate the delivery of online psychotherapy at a low cost and allows therapy to be highly accessible and anonymous [9]. There have been an increasing number of studies examining the effectiveness of Web-based cognitive bias modification interventions. In 2015, Wittekind et al evaluated an online avoidance retraining intervention for 257 individuals with a tobacco use disorder; and demonstrated that avoidance bias retraining was associated with a significant reduction in the number of cigarettes smoked and the cravings to smoke [10]. In 2017, Cougle et al conducted a trial which evaluated an online interpretation bias modification program for hostility with 58 individuals [11]. They found that the online intervention led to a greater reduction in interpretative bias compared to the control group. These trials have demonstrated the feasibility and effectiveness of Web-based bias modifications. Further advancement in technology, coupled with increased ownership of mobile devices, has led to there being increasingly more interventions that use mobile technologies (mHealth). Based on the published literature to date, there are several studies that have evaluated the potential and effectiveness of mHealth bias modification. Of the studies, seven reported that mHealth bias modification was effective for participants with a variety of disorders, namely insomnia, alcohol, tobacco use, or social anxiety disorders [12-18]. mHealth technologies are increasingly being harnessed for the delivery of bias modification, as mobile technologies allow for the training to be conducted in diverse locations, thus helping in the generalization of clinical benefits [19]. In addition, such technologies help to increase accessibility to the intervention and aid in the reduction of costs associated with treatment. The use of mobile technologies in the delivery of bias modification interventions could help to improve outcomes, in that it helps to increase the frequency of training [19]. mHealth technologies have an advantage over existing Web-based versions, given that web interventions would require individuals to be consistently connected to the internet to undertake the bias modification task. There is also the potential to couple the bias modification with other sensors in the mobile phone, such that users will be prompted to engage in training tasks to help them with their symptoms if they are deemed to be in a high-risk locality [20].

While the existing mHealth studies have demonstrated the feasibility of such technologies, most of these studies are limited to a description of the conventional bias modification methodology that has been adopted. None of the previous studies have shared the platform that they have used in the development of their cognitive bias task, thus limiting the replicability of the intervention. Most studies are limited to a description of the conventional task paradigm that have been adopted, for example, in the study reported by Clarke et al in 2016, it was reported that their intervention task was based on the conventional attentional probe training paradigm [12]. In the supplementary material, details of the words that were utilized for their task were provided. Whilst this allows for replicability to a certain extent, a number of resources are needed to develop a mobile version of the platform to deliver cognitive bias modifcation. Hence, it is thus of great import to have a common platform that could easily facilitate the design and customization of such interventions for a variety of psychiatric disorders. As a start, we will describe the design of a research platform that allows the customization of bias modification interventions for a range of substance use disorders.

## Methods

### Overview of Research Platform

**Figure 1:**
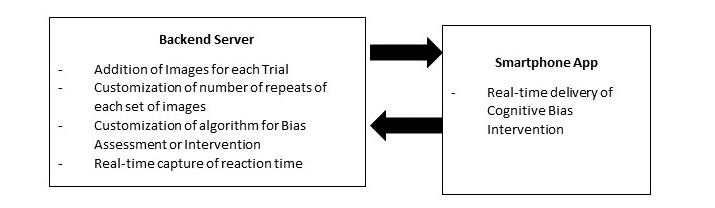
Overview of the research platform for the cognitive bias intervention for substance use disorders.

A multidisciplinary team, comprising of 2 psychiatrists specializing in addiction psychiatry, a psychologist with prior expertise on attentional bias modification, and a computer engineer were involved in the development of the research platform. It was decided that the platform would comprise of a mobile phone version of the cognitive bias task (as an example, the visual probe task), which is controlled both by a server that could customize the algorithm for the tasks and collate the reaction time data in realtime (Figure 1). The server would also allow the researcher to program the specific set of images that will be present in the task. The mobile phone app would synchronize with the backend server in realtime.

The mobile phone app that will be created needs to be modelled against the conventional visual probe task approach [21]. Based on the conventional approach, in the assessment phase, individuals would be presented with a fixation cross upon commencement. After that, both a neutral and non-neutral stimulus would be presented simultaneously. Both of these stimuli would disappear and a probe (usually in the form of an asterisk) would replace either stimulis. Individuals are required to indicate the position of the asterisk by indicating a response within a predetermined time. In the bias assessment phase, the asterisk would replace either stimulis equally (50% of the time). However, in the bias modification phase, the asterisk would replace the neutral stimulus all the time (100% of the time). Individuals with biases would spend more time engaging with the stimuli and have difficulties with disengaging after. This will be reflected in their increased reaction time towards identifying the exact locality of the probe (asterisk).

**Figure 2:**
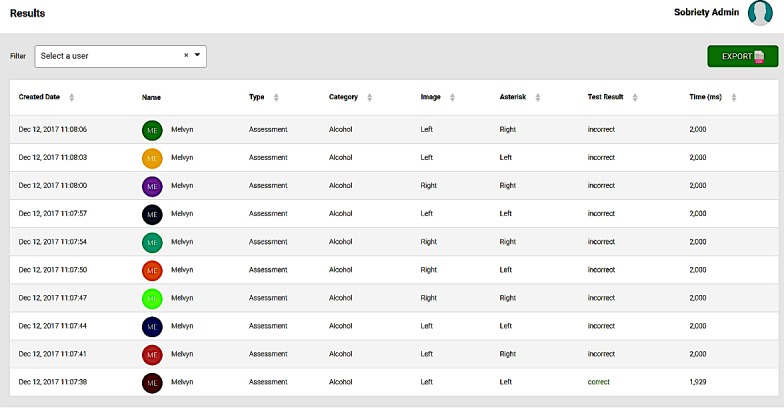
Sample dataset of reaction time data collated by the server.

**Figure 3:**
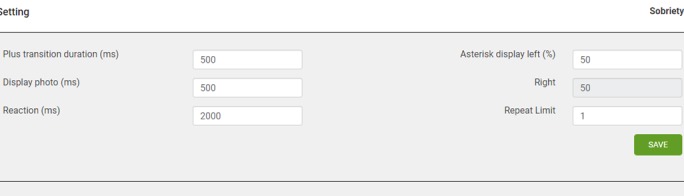
Ability of the server to customize the algorithm for the Visual Probe task.

The graphic user interface will be similar across the different substance disorders. The server will allow for the collation of reaction time data, as well as other variables that are essential when using reaction time to compute the presence of attentional biases. These variables include the positions of the probes, the positions of the substance-related stimuli, and whether the participant has indicated a correct response. Figure 2 provides an overview of the sample dataset that is collated by the server. In addition, the server has been programed with a specific algorithm, such that it enables the control of the frequencies of the probe replacing the substance stimuli (whether 50% or 100%). The research platform would also allow the researchers to vary the number of repeats of a particular set of images. Figure 3 provides an overview of how the server is able to control these parameters.

### Development of Research Platform

The cognitive bias modification app was developed using an open-source cross-platform gaming software, React Native. The development team decided to utilize this approach for several reasons. The integration of an open-source approach would ensure that the intervention is compatible and could be utilized across a variety of platforms, without any differences between platforms. Thus, participants are able to undertake the task on their mobile phone devices, as well as on a normal computer. The gaming platform that has been used also allows for the future incorporation of gamification elements, as gamification has been shown to help increase motivation for bias modification [22]. In addition, the current platform is verstaile and allows for scaling or modification of the entire progam to be used for a different disorder. This would drastically reduce the time and cost associated with development, while ensuring that the timings of the conventional paradigms are retained.

## Results

Multimedia Appendix 1 contains a video demonstrating the operation of the app, as well as a sample dataset of the reaction times captured by the app (Multimedia Appendix 2).

## Discussion

### Principal Findings

The current design could be utilized for cognitive bias modification across a spectrum of disorders and is not limited to just one disorder. This research platform could be adopted by various cognitive bias modification interventions and the usage of this platform would potentially drive down the costs associated with the development of such mHealth interventions. This is congruent with the recommendation made in the study published by Zhang et al for the usage of low-cost methodologies in app development [23]. The framework we have developed could be easily distributed and fellow researchers could make use of the created platform for their own interventions. While we have presented and discussed evidence mainly for substance use disorder, cognitive bias modification is also efficacious for other disorders, such as social anxiety disorders [24]. As compared to the conventional method, or even Web-based interventions, the mobile phone bias modification intervention would imply that individual users are no longer confined to a laboratory environment to receive the intervention. Individuals can make use of the interventions in their naturalistic environments, and the app could also be coupled with geolocation services intrinsic to the mobile phone. By doing so, this would facilitate the delivery of timely interventions when individuals are in their high-risk locality, when they are most prone to a relapse.

Furthermore, there has been growing interest in consideration of incorporating of serious game elements to conventional cognitive bias tasks [7], and subsequently a study has been published which highlighted several possible gamification strategies [22]. A variety of serious games strategies have been proposed, which include the addition of game elements, the intrinsic integration with the evidence-based task as a basis, intrinsic integration leaving the evidence base task intact, or the addition of a game shell around the original evidence base task [22]. These four different mechanisms could easily be achievable in the current design, given that the design of the current mobile phone based attentional bias modification paradigm has been based on a gaming engine. Whilst most gamification techniques seek to increase an individual’s motivation to continue with a task, it is important to recognize that the consequences of the game do have an impact on gaming behaviors [19]. In our case, the pairing of an aversive consequences in the game play might help to enhance our aim of decreasing individuals’ typical behavior towards rewarding stimuli.

### Conclusions

In conclusion, we have shared a research platform for the design of a mobile phone version of a bias modification task, which could be easily modified to be applied to a spectrum of disorders. It will be of value for future research to utilize the above platform and compare the efficacy of mHealth approaches, such as the one described in this study, with conventional Web-based approaches in the delivery of cognitive bias modification interventions. Additionally, the use of a gaming platform in the current design opens future opportunities to consider the addition of serious gaming elements to enhance the appeal of these tasks.

## Appendix

Multimedia Appendix 1 Demonstration of the research platform app.

Multimedia Appendix 2 Sample dataset of reaction times.

## Abbreviations

eHealth: electronic health
mHealth: mobile health
